# Childhood Emotional Abuse Moderates Associations Among Corticomotor White Matter Structure and Stress Neuromodulators in Women With and Without Depression

**DOI:** 10.3389/fnins.2018.00256

**Published:** 2018-04-23

**Authors:** Carlton P. Frost, M. Elizabeth Meyerand, Rasmus M. Birn, Roxanne M. Hoks, Erin C. Walsh, Heather C. Abercrombie

**Affiliations:** ^1^Department of Psychiatry, University of Wisconsin-Madison, Madison, WI, United States; ^2^Department of Medical Physics, University of Wisconsin-Madison, Madison, WI, United States; ^3^Department of Biomedical Engineering, University of Wisconsin-Madison, Madison, WI, United States; ^4^Department of Psychiatry, University of North Carolina at Chapel Hill, Chapel Hill, NC, United States

**Keywords:** cortisol, sympathetic nervous system, hypothalamic pituitary adrenal axis, corticomotor system, emotional abuse, depression, diffusion tensor imaging, tract-based spatial statistics

## Abstract

Adverse caregiving during development can produce long-lasting changes to neural, endocrine, and behavioral responses to stress, and is strongly related to elevated risk of adult psychopathology. While prior experience of adversity is associated with altered sympathetic nervous system (SNS) and hypothalamic-pituitary-adrenal (HPA) axis activity, the underlying neural pathways are not completely understood. In a double-blind crossover study, we used diffusion tensor imaging (DTI) to examine whether variation in white matter structure predicts differences in HPA-SNS interactions as a function of early adversity. Participants included 74 women who exhibited a wide range of depression severity and/or childhood emotional abuse (EA). Participants attended two experimental sessions during which they were administered 20 mg cortisol (CORT) or placebo and after 90 min, viewed emotionally laden pictures while undergoing MRI scanning. Immediately after emotional picture-viewing, we collected salivary alpha-amylase (sAA) to index SNS activation. We tested whether EA moderated the relation between fractional anisotropy (FA), a measure of white matter fiber structure, and sAA. In the placebo condition, for participants with minimal history of EA, higher FA in corticomotor projections was negatively correlated with sAA, whereas in participants with severe EA, the correlation was trending in the opposite direction. Following CORT administration, FA and sAA were not related, suggesting that SNS tone during acute cortisol elevation may depend on neural pathways other than corticomotor projections. The results suggest that at baseline—though not during cortisol elevation—increased FA in these tracts is associated with lower levels of SNS activity in women with minimal EA, but not in women with severe EA. These findings provide evidence that corticomotor projections may be a key component of altered neural circuitry in adults with history of maltreatment, and may be related to alterations in stress neuromodulators in psychopathology.

## Introduction

Experience of adverse caregiving in childhood is a risk factor for a variety of psychopathologies in adulthood, including major depressive disorder (MDD) and posttraumatic stress disorder (PTSD). Studies in both humans and animals show that responses to acute stress are shaped by the history of the organism (Pavlides et al., 2002; Alfarez et al., 2003; Ellis et al., 2005; Joëls and Krugers, 2007; Meewisse et al., 2007; Ehring et al., 2008). Prior adversity is at times associated with high sympathetic nervous system (SNS) response (featuring the systemic release of the catecholamine epinephrine in the medulla of the adrenal gland; Otte et al., 2005), and low hypothalamic-pituitary-adrenal (HPA) response (featuring systemic release of glucocorticoids [GCs], primarily cortisol in primates or corticosterone in rodents) to acute stress (Resnick et al., 1995; McFarlane et al., 1997; Walsh et al., 2013; Drury et al., 2016). Furthermore, it has been hypothesized that the balance between SNS and HPA responses to acute stressors is altered in individuals previously exposed to chronic and/or severe stress, so that cortisol signaling does not sufficiently contain the SNS response (Yehuda et al., 1998). The neural pathways supporting such alterations in peripheral stress physiology in individuals previously exposed to adversity are unknown.

Functional activity in a variety of brain regions and circuits, such as hippocampus, amygdala, and fronto-limbic circuitry, regulates and is modulated by stress neuromodulators such as catecholamines and GCs (Roozendaal et al., 2009; Ulrich-Lai and Herman, 2009; Hermans et al., 2014; Vogel et al., 2016). More recent research (Dum et al., 2016; Abercrombie et al., 2018) in humans and nonhuman primates has indicated a possible role for cortical premotor areas including supplementary motor area (SMA), cingulate motor areas (CMAs), and premotor cortex (PMC) in neural response to and control of stress neuromodulators. For instance, anatomical tracing in Cebus monkeys shows these areas project densely to spinal circuits innervating sympathetic preganglionic cells, which terminate in adrenal medulla, likely modulating peripheral SNS activation (Dum et al., 2016).

In addition, dorsomedial frontal cortical areas, which share substantial overlap with these premotor regions, are known substrates for GC modulation of neural activation and HPA tone (McEwen et al., 1986; Diorio et al., 1993; Radley et al., 2006). Such a pathway could constitute a potential interface by which trauma and adversity may give rise to altered HPA/SNS relationships. Indeed, numerous studies have found that patterns of dorsomedial prefrontal activation and connectivity during emotion regulation differ in individuals with history of maltreatment (McLaughlin et al., 2015; McCrory et al., 2017) and may also distinguish between individuals resilient to or at risk of psychopathology related to early adversity (Herringa, 2017).

To further elucidate the neural pathways by which adversity relates to alterations in stress response systems, we tested relations between measures of structural connectivity and SNS activation in a sample of women with varying history of childhood emotional abuse (EA) and psychopathology. We further tested how exogenous cortisol altered these relationships. The use of exogenous cortisol permits inferences about the effects of cortisol *per se*, rather than the effects of elevations in endogenous cortisol elicited by a potent, social-evaluative stressor (Dickerson and Kemeny, 2004). We pharmacologically manipulated cortisol (CORT) vs. placebo before participants viewed emotionally evocative pictures, and measured salivary alpha-amylase (sAA) following the task to index SNS activation. Alpha-amylase is an enzyme whose salivary concentration can be used as an index of SNS activity, such as when detecting autonomic dysregulation or treatment response (Nater and Rohleder, 2009). Brain structure was assessed using T1- and diffusion-weighted magnetic resonance imaging. Using functional magnetic resonance imaging (fMRI) in this sample, we previously reported that childhood EA moderated the effects of CORT on activation in SMA and adjacent premotor cortical areas, and that this activation was related to sAA, an index of sympathetic adrenal-medullary output. Because descending tracts such as corticospinal tract (CST) likely carry corticomotor axons en route to sympathetic preganglionic cells (Dum et al., 2016), we therefore hypothesized that CORT's effects on sAA would be related to their white matter structure, and that this relationship would be moderated by history of childhood EA.

## Materials and methods

### Participants

Participants were women aged 18–45 with varying depression severity and history of childhood EA. We did not specifically recruit women with anxiety disorders or PTSD, but these were not exclusionary (Table 1; full eligibility criteria in Supplementary Materials). Of 85 eligible participants, 80 completed the study and full data were available for 75. Data was lost to experimenter error (1 participant), scanner malfunction (1 participant), poor image quality (2 participants), and a medical condition (1 participant), and 1 participant was excluded as an extreme outlier during data analysis (see next section), bringing the final N to 74. The study protocol was approved by the University of Wisconsin Health Sciences IRB. Participants provided written informed consent and were paid for participation.

**Table 1:** Demographic and clinical characteristics.

**Characteristics**	**CTQ emotional abuse groups**		
	**Minimal (*n* = 46)**	**Moderate (*n* = 14)**	**Severe (*n* = 14)**
Age, years^a^	26.1 ± 6.4	31.4 ± 7.1	28.1 ± 8.0
Lifetime depressive disorder	23 (50.0)	9 (64.3)	13 (92.9)
Current depressive disorder	12 (26.1)	7 (50.0)	13 (92.9)
Current anxiety disorder	12 (26.1)	6 (42.9)	9 (64.3)
Current PTSD	0	3 (21.4)	6 (42.9)
Race^b^^,^^c^			
White	34 (73.9)	9 (64.3)	12 (85.7)
Asian	8 (17.4)	3 (21.4)	2 (14.2)
African American	3 (6.5)	1 (7.1)	0
Unknown	1 (2.2)	1 (7.1)	0
Ethnicity^b^			
Hispanic/Latina	4 (8.7)	2 (14.3)	0
Not Hispanic/Latina	42 (91.3)	11 (78.6)	14 (100)
Unknown	0	1 (7.1)	0
Education level^d^	4.4 ± 1.4	5.2 ± 1.1	4.9 ± 1.3
Childhood caregivers' education level^d^	4.5 ± 1.7	4.9 ± 1.2	5.1 ± 1.7

*Values are mean ± SD, or n (groupwise %). CTQ, Childhood Trauma Questionnaire; PTSD, posttraumatic stress disorder*.

a *There was a small but significant group difference in age, F_(2, 73)_ = 3.25, p = 0.04*.

b *Chi-squared tests confirmed the CTQ Emotional Abuse groups did not significantly differ by racial or ethnic composition, p's > 0.34*.

c *Because of rounding, percentages may not total 100*.

d *Education categories: 1, Less than high school; 2, High school diploma or equivalent (i.e., GED); 3, Some college, no degree; 4, Associate's degree; 5, Bachelor's degree; 6, Master's degree; 7, Doctoral degree*.

### Measuring childhood EA and depression severity

Childhood EA was retrospectively assessed using the Emotional Abuse subscale of the Childhood Trauma Questionnaire (CTQ), a well-validated instrument that can be used to measure aversive caregiving continuously or by categorizing participants into groups using standard cut scores (Bernstein et al., 2003). In the final sample, 14 women experienced moderate-to-extreme (“severe”), 14 experienced low-to-moderate (“moderate”), and 46 experienced none-to-minimal (“minimal”) childhood EA. Timing of EA was assessed using a life history calendar (Caspi et al., 1996), which confirmed that all women endorsing EA experienced abuse prior to menarche, and many experienced ongoing EA from early childhood through adolescence.

Consistent with the NIMH Research Doman Criteria (RDoC) framework (Insel, 2014), which emphasizes the continuous nature of psychiatric disorders, we recruited women with a range of depression, anxiety, and PTSD severity. Psychopathology was assessed using the SCID-I/P for DSM-IV-TR with additional questions to assess DSM-5 criteria (First et al., 2002). Some participants experienced clinically significant anxiety (36%) and PTSD (12%); these disorders were comorbid with depression, for which we specifically recruited and which was more prevalent in our sample (61% lifetime, 43% current; Table 1). Depression severity was indexed using the average of Beck Depression Inventory-II (BDI-II) scores across two experimental sessions (Beck et al., 1996). A square-root transformation was applied to reduce negative skew and undue influence of extreme BDI-II scores as in prior research (van Minnen et al., 2005; Roelofs et al., 2013). In scatter plots, scores are back-transformed to preserve initial range.

Although we sought to recruit a sample in which EA and depressive symptoms were not entirely overlapping (Table 1), EA was nevertheless strongly associated with adult depression: the correlation in this sample is *r*_(73)_ = 0.45, *p* < 0.01. One participant's scores on the BDI-II and EA subscale of the CTQ were 0 and 25, which are respectively the lowest and highest possible scores. These values exerted extreme and anomalous influence on results: standardized residuals > 2, Cook's distance > 4 times mean, leverage >> 2*p*/*n*, thus meeting criteria for outlier identification proposed by Rawlings et al. (1998). This was the only participant in the sample who concurrently reported extreme EA and no depressive symptomatology. Thus, this participant skewed statistical distributions and was ultimately classified as an outlier and excluded from analysis. For discussion, see Limitations Section.

### Procedure

Cortisol levels were manipulated by administering 20 mg oral hydrocortisone (i.e., exogenous cortisol; CORT). In a separate experimental session, participants were given an identically appearing placebo capsule. The CORT and placebo sessions were conducted in the late afternoon into the evening (beginning at 4:15 PM) and were typically separated by 1 week (minimum of 5 days). The order of drug administration was randomized and double-blinded. Neuroimaging (diffusion tensor imaging [DTI] and fMRI) was conducted during both visits. fMRI data are described elsewhere (Abercrombie et al., 2018).

### SNS activation during CORT vs. placebo

**Figure 1:**
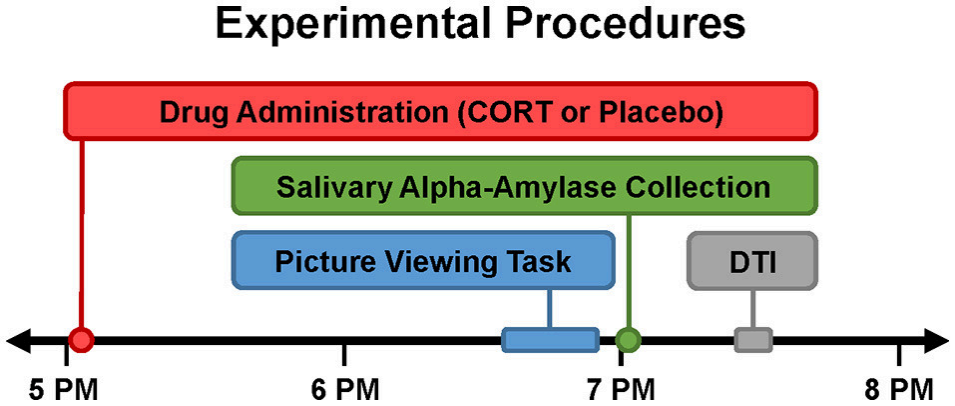
Timing of experimental procedures. Study participation entailed two experimental sessions, which were typically separated by 1 week (minimum of 5 days). All experimental sessions were conducted late in the day (beginning at 4:15 PM) when endogenous cortisol levels are relatively low. Drug (20 mg oral hydrocortisone [CORT] or placebo) was administered slightly after 5PM. Order of drug administration across the two sessions was randomized and double-blinded. Approximately 90 min after drug administration, participants viewed emotional pictures during fMRI (described elsewhere; Abercrombie et al., 2018). Salivary alpha-amylase (sAA) was collected immediately after the emotional picture viewing task to index sympathetic nervous system (SNS) activity during CORT vs. placebo. Diffusion tensor imaging (DTI) was conducted to assess white matter structure.

To index SNS activation in the context of an emotionally evocative experience, sAA was measured immediately after participants completed an emotional picture viewing task, which occurred 90 min after drug administration during fMRI (Figure 1). Participants viewed one of two sets of psychometrically matched pictures from the International Affective Picture System (IAPS; Lang et al., 2008) during each session.

For measurement of sAA and salivary cortisol levels, saliva samples were collected from participants throughout each session with Salivettes (Sarstedt, Nümbrecht, Germany), according to previously-described guidelines (Rohleder and Nater, 2009). sAA concentrations were assayed with an enzyme kinetic method (e.g., Bosch et al., 2003). Intra- and inter-assay coefficients of variation were below 11%. Cortisol concentrations were measured with a high sensitivity chemiluminescence immunoassay (IBL International, Hamburg, Germany; intra- and inter-assay coefficients of variation below 8%) to confirm that post-drug administration cortisol levels did not vary by group (see Table 2). Log-transformation was used to normalize distributions for sAA and salivary cortisol (Rohleder and Nater, 2009).

**Table 2:** Salivary analytes by childhood emotional abuse (EA) severity and drug.

**Measure**	**CTQ emotional abuse groups**		
	**Minimal (*n* = 46)**	**Moderate (*n* = 14)**	**Severe (*n* = 14)**
**sAA LEVELS, U/mL**			
Placebo	191.8 ± 155.9	172.9 ± 155.6	236.6 ± 193.8
CORT	166.0 ± 115.1	221.7 ± 225.9	187.4 ± 134.1
**SALIVARY CORTISOL LEVELS, nmol/L**			
Placebo	1.3 ± 1.6	1.7 ± 2.1	1.3 ± 0.7
CORT	55.4 ± 33.6	54.2 ± 32.7	50.6 ± 41.9

*Values are mean ± SD. CORT, cortisol administration; CTQ, Childhood Trauma Questionnaire; sAA, salivary alpha-amylase*.

*Raw values for sAA and cortisol are shown in the table. Log-transformed values were used for analyses to normalize distributions. Neither sAA nor cortisol showed main effects or interaction for Drug (Placebo vs. CORT) or CTQ Emotional Abuse (EA), p's > 0.33*.

### Image collection and preprocessing

Brain images were collected using a 3T General Electric MRI scanner (Discovery MR750; GE Medical Systems, Waukesha, WI) equipped with an 8 channel RF coil (GE Healthcare, Waukesha, WI). Structural data were acquired using a T1-weighted BRAVO pulse sequence (TI:450 ms, TR/TE/flip:8.16 ms/3.2 ms/12°, matrix:256 × 256 × 160, FOV:215.6 mm, slice thickness:1 mm). Diffusion-weighted data were acquired using a 2D echo planar imaging (EPI) sequence with ASSET parallel imaging at a geometric reduction factor of 2 in order to correct magnetic field inhomogeneities (TR/TE/flip:8,000 ms/66.2 ms/90°, matrix:128 × 128 × 76, FOV:256 mm, slice thickness:2 mm). Each axial slice was encoded with 48 directions, b = 1,000 s/mm^2^, and eight b0 images. Field maps to correct for magnetic field distortions were also collected. Images were corrected for eddy current and fieldmap distortions, and skull-stripped (FMRIB Software Library, FSL; Smith et al., 2004). Images from the first scan session were used for analysis. Tensors were fitted with nonlinear optimization, constrained to be positive semi-definite using Camino (camino.cs.ucl.ac.uk; Cook et al., 2006). Tensor images were normalized, and scalar maps for fractional anisotropy (FA) and axial, radial, and mean diffusivity were calculated, using Diffusion Tensor Imaging ToolKit (DTI-TK, dti-tk.sourceforge.net).

To create seed regions with which to virtually dissect tracts of interest, T1 images were processed using FreeSurfer's automatic recon-all pipeline including preprocessing steps (motion correction, intensity normalization, affine registration to Talairach atlas, brain extraction), linear registration to a Gaussian classifier array for automatic subcortical segmentation (v5.3, https://surfer.nmr.mgh.harvard.edu; Fischl et al., 2002), and estimation and parcellation of cortical surfaces into functional and anatomical compartments (Desikan et al., 2006; Destrieux et al., 2010).

### Data analysis

#### CORT's effects on SNS activation in relation to EA

Proc GLM in SAS (SAS version 9.4, Cary, NC) was used to test (1) whether there was a main effect of Drug (CORT vs. Placebo) on sAA, and (2) whether effects of Drug on sAA differed with respect to EA. Analyses were conducted on log-transformed (i.e., normalized) values of sAA.

#### DTI

##### Tract-based spatial statistics (TBSS).

Individual subject maps were co-registered to a bootstrapped group template, and warps to that template were computed, by implementing DTI-TK (Zhang et al., 2007) using a custom tool (Diffusion Image Processing and Analysis, DIPA; github.com/pegasus-isi/dipa-workflow) implemented through Condor, developed by the Center for High Throughput Computing at the University of Wisconsin-Madison & Wisconsin Institutes for Discovery (chtc.cs.wisc.edu). Whole-brain, voxelwise statistical analysis of white matter structure was carried out using the TBSS method in FSL, which entails the creation of a white matter skeleton along which each subject's projected data are then analyzed (Smith et al., 2006).

To elucidate the relationship of childhood EA and adult white matter structure, general linear models were constructed accounting for age as a nuisance regressor and for depression severity, and were entered into FSL's Randomize program. Randomize performs nonparametric permutation to allow inference on data whose null distribution is not known (Winkler et al., 2014). Family-wise error rates were corrected using threshold-free cluster enhancement, which assigns voxel-wise values based on cluster-like local spatial agreement (Smith and Nichols, 2009). Next, we tested whether EA moderated the relationship between white matter structure (FA) and effects of CORT (vs. Placebo) on sAA. The difference in sAA between the CORT and Placebo days (sAA_CORT−Placebo_) therefore represented our experimental variable, and an interaction term specified whether EA moderated these effects. To confirm that depression severity did not also moderate the relationship between sAA_CORT−Placebo_ and FA, a model correcting for age and including terms for BDI, sAA_CORT−Placebo_, and their interaction was also entered into Randomize.

Once clusters of interest were identified, they were warped from template space to participant space and mean FA values over the cluster were extracted from corresponding regions in each participant. For *post hoc* analyses, these FA values were entered into ANCOVA using SAS to characterize the relations between FA and sAA, and differences in these relations across EA groups.

##### Deterministic tractography

TBSS-derived clusters often include fiber bundles from multiple tracts of interest, which may be disambiguated to aid in interpreting their functional significance. To anatomically validate the affected tracts, deterministic tractography was performed in subjects' native space using Camino and visualized in TrackVis (trackvis.org; Wang et al., 2007). Significant voxels were used as seeds for streamline tractography using the fiber assignment by continuous tracking (FACT) algorithm. Streamlines were terminated at voxels with FA values less than 0.1 or when local curvature exceeded 60°. Among streamlines passing through the significant white matter clusters, tract membership was determined using anatomical landmarks and subject-specific segmentation-derived seed regions from FreeSurfer, as described in the Results. In general, when referring to “clusters,” we mean voxels found significant in the TBSS model; by “tracts” we mean those a priori, anatomically-identifiable tracts whose streamlines were found to pass through the clusters of interest.

## Results

### Participants could not distinguish between drug conditions

Consistent with prior research (Abercrombie et al., 2005, 2011; Wirth et al., 2011), participants did not perform above chance in distinguishing between CORT and Placebo conditions: when asked on each of the first and second visits what they thought had been administered, 60 and 67% of participants said they did not know, respectively, and of those that guessed, accuracies were 52 and 38%, *p*'s > 0.05.

### Effects of CORT on SNS activation

No main effect of Drug (CORT vs. Placebo) on sAA was observed, *F*_(1, 74)_ = 0.09, *p* = 0.76 (Table 2). In addition, sAA level was not related to EA in either the Placebo or CORT condition or its interaction with Drug, *p*'s > 0.18. sAA was also not related to depression or its interaction with Drug, *p*'s > 0.59.

### SNS activation and white matter structure

**Figure 2:**
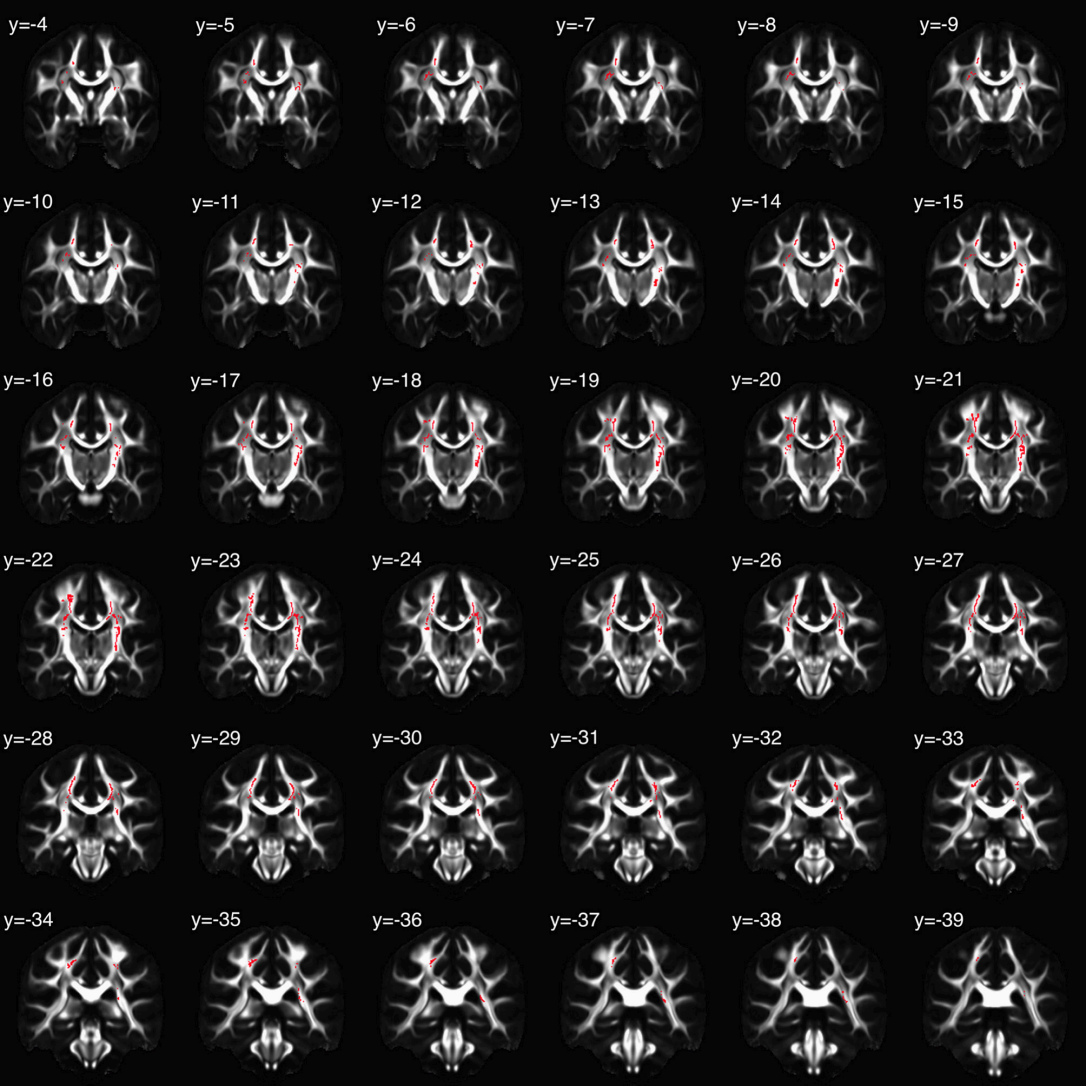
Clusters in which the relation between fractional anisotropy (FA) and salivary alpha-amylase (sAA) was moderated by childhood emotional abuse (EA). Coronal slices are presented in montage, beginning with *y* = −4 in MNI space, radiological orientation (i.e., participants' right is on viewer's left).

**Figure 3:**
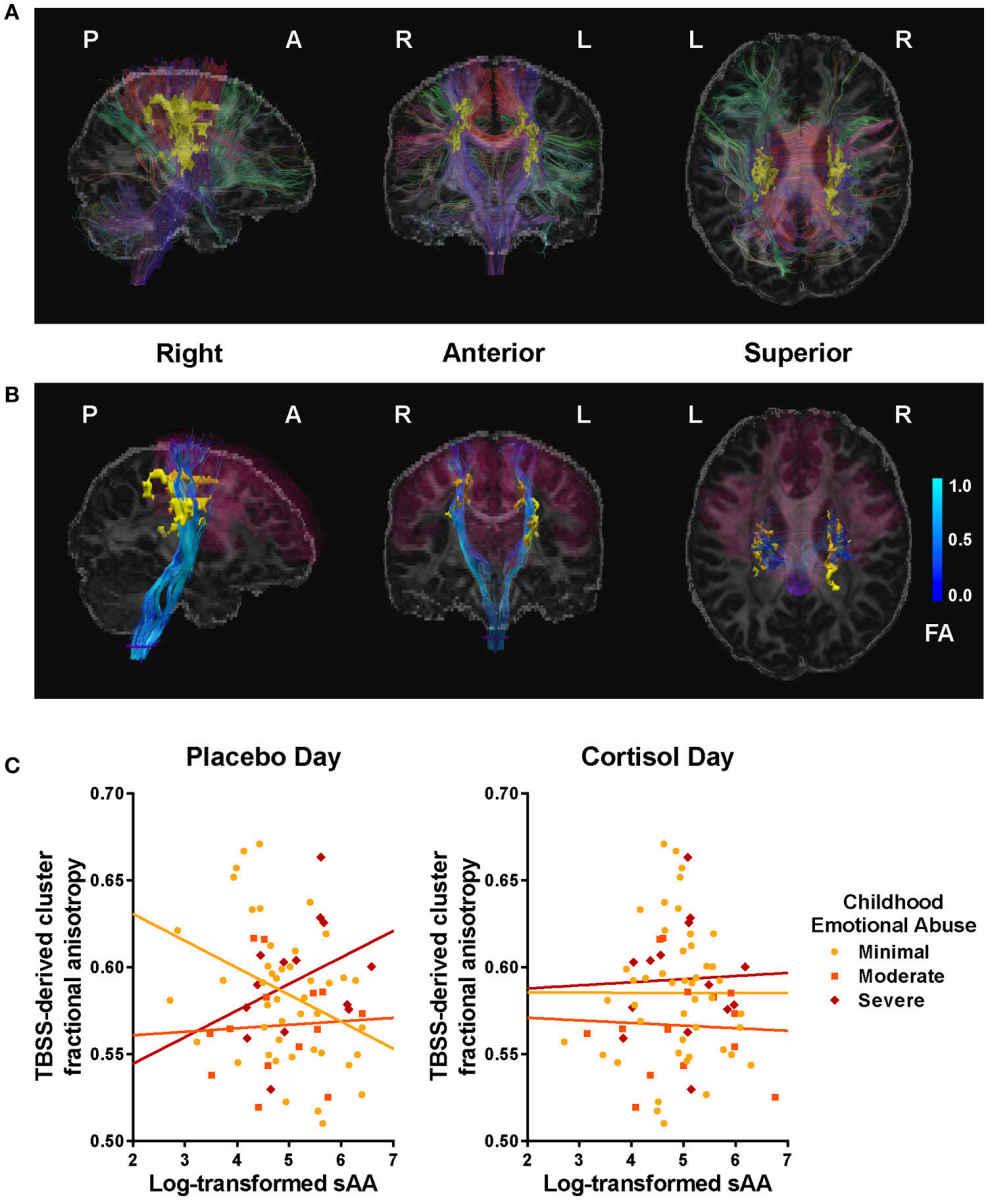
Clusters and tracts in which the relation between fractional anisotropy (FA) and salivary alpha-amylase (sAA) was moderated by childhood emotional abuse (EA). **(A)** Tract-based spatial statistics (TBSS)-derived clusters (yellow) significantly associated with EA × sAA_CORT−Placebo_ interaction, illustrated with a representative participant, and fiber tracts intersecting clusters (color indicates direction at fiber midpoint: red fibers run primarily left-right, green anterior-posterior, blue superior-inferior). See Table 3 for listing of significant clusters**. (B)** Projection fibers (blue) originating from corticomotor regions (magenta, derived from FreeSurfer segmentation: precentral gyrus, paracentral gyrus, posterior & caudal anterior cingulate gyrus & sulcus, superior frontal gyrus, and caudal middle frontal gyrus), passing through TBSS-derived cluster (yellow), and reaching spinal cord (purple) after virtual dissection using FreeSurfer segmentation. Brighter blue indicates higher FA. **(C)** Scatter plots for sAA and cluster FA, plotted by EA. At left, Placebo-day correlations; at right, CORT-day correlations. EA moderated the association between cluster FA and sAA levels during placebo administration but not during CORT, *F*_(6,74)_ = 3.90, *p* < 0.01.

TBSS-derived clusters of interest shown in Figures 2 and 3, and Table 3 represent regions in which FA was significantly associated with EA, sAA_CORT−Placebo_, or their interaction. Clusters associated with the interaction were centered in bilateral white matter tracts ventral to motor and premotor cortices at the level of the centrum semiovale (Figure 3A), at the intersection of the CST, corpus callosum (CC), thalamic radiations/corona radiata (CR), and inferior fronto-occipital fasciculus (IFOF). Corticomotor projections were identified as intersecting at least one of precentral, paracentral, caudal middle frontal, superior frontal, posterior cingulate or caudal anterior cingulate gyri, and reaching spinal cord. In addition, we adapted landmarks from prior studies (e.g., Bleyenheuft et al., 2007) to identify fibers belonging to CST proper (Figure S1). IFOF fibers terminated anteriorly in precentral, inferior or middle frontal gyri and posteriorly in parietal or superior temporal areas. CR terminated in thalamus.

**Table 3:** Childhood emotional abuse (EA), salivary alpha-amylase (sAA), and fractional anisotropy (FA).

**Cluster**	**Volume (voxels)**	**Coordinate (peak)**	**Coordinate (CM)**	**T-Stat (peak)**	***P-*value (corrected)**
**EA**					
None	–	–	–	–	–
**sAA_CORT−Placebo_**					
Left corticofugal tracts	2,373	(−28,−3,4)	(−23,−4,13)	3.74	0.01
Right corticofugal tracts	2,136	(20,−3,34)	(22,−4,15)	4.14	0.01
Left mid superior CC	114	(−18,20,27)	(−17,22,16)	3.33	0.05
Left centrum semiovale	17	(−27,4,27)	(−27,4,27)	3.71	0.05
Left superior CR	13	(−24,21,16)	(−24,21,16)	3.34	0.05
EA ^*^ sAA_CORT−Placebo_					
Left corticomotor tracts	1,341	(−28,−3,4)	(−25,−4,17)	3.56	0.02
Right corticomotor tracts	1,307	(18,−3,34)	(21,−5,24)	4.22	0.03

*CM, center of mass; CC, corpus callosum; CR, corona radiata; EA, Emotional Abuse subscale of the Childhood Trauma Questionnaire; sAA_CORT−Placebo_, the difference in salivary alpha-amylase between the CORT and Placebo days*.

*Significant clusters associated with model factors*.

The relation between tractographic “streamline” count and anatomical fiber count is highly conditional, and should be understood as a qualitative rather than quantitative tool (Jones et al., 2013b), but may offer a very coarse measure of comparative connectivity. In all participants examined, a large quantity of streamlines belonging to CST and other motor cortical projections to spinal cord were readily identified, of which the overwhelming majority traversed the cluster of interest. Approximately half or fewer of those streamlines belonging to IFOF did so, and those that did were generally limited to the tract's superior extent (i.e., terminating in superior parietal areas). CR terminating in thalamus varied in cluster membership.

Posthoc analyses were conducted to disentangle the significant TBSS interaction. Using mean cluster FA, we found that the correlation between sAA_CORT−Placebo_ and FA differed across EA groups, *F*_(6, 74)_ = 3.90, *p* < 0.01, *R*^2^ = 0.26. This effect was driven by Placebo-day differences in slope, *F*_(6, 74)_ = 3.02, *p* = 0.01, *R*^2^ = 0.21 (Figure 3C), such that for women with minimal childhood EA, post-task sAA levels were negatively associated with higher cluster FA (*r* = −0.30, *p* = 0.02), but for women with history of severe EA the association was positive though did not reach significance (*r* = 0.37, *p* = 0.20). Cluster FA was unrelated to CORT-day sAA and its interaction with EA, *p*'s > 0.19 (Table 3). Further investigation showed that effects were mainly driven by opposing group-wise relations between sAA and radial diffusivity; axial and mean diffusivity were unrelated to sAA and EA.

FA was associated with depression severity in a cluster localized to the anterior CC (Table S1, Figure S2), but was not associated with sAA or the interaction between depression severity and sAA.

## Discussion

Consistent with our hypothesis, we found that the structural properties of bilateral corticomotor projections were related to SNS activation following exposure to emotional stimuli. Specifically, we found that the relation of corticomotor FA to SNS activation differed among women with history of severe childhood emotional abuse vs. those without, and CORT administration eliminated these differences. Depression severity did not moderate these effects. These findings provide evidence that these corticomotor projections may constitute an important pathway underlying alterations in stress neuromodulators in individuals with a history of abuse.

### Differences in FA are likely driven by corticomotor projections

Streamlines belonging to a variety of tracts traversed the clusters extracted from the TBSS analysis. Although tractography cannot definitively confirm or exclude the contribution of individual tracts to detectable FA effects, it can aid in interpreting likely drivers. In all participants examined, the most reliable corticofugal projections linked brainstem and spinal cord to sensorimotor areas (Figure S3). Using anatomical landmarks to positively identify its descending course, CST streamlines were identified as traversing the clusters of interest more reliably and in greater proportion than other tracts: of streamlines belonging to CST, over 90% were identified in multiple participants. Most such streamlines appeared to terminate on the dorsal surface of precentral and superior frontal gyri, although precise departures of fibers from a larger tract can be difficult to resolve (e.g., Jones et al., 2013a). Other premotor and sensorimotor projections reaching spinal cord showed similarly high rates of capture by our clusters.

Although a majority of streamlines of the mid-to-posterior CC traversed the clusters, the structure's interhemispheric extent—one of the most robust and undiluted white matter tracts in the brain—did not reach significance, and bilaterally the significant clusters extend well inferior to the main CC projections. This suggests that interhemispheric connectivity, while possibly involved, likely is not the principal driver our effects.

A variable proportion, typically half or fewer, of IFOF streamlines intersected the clusters, and those generally in dorsal parietal areas, in contrast to more ventral tracts such as those identified in Rodrigo et al. (2016). However, future research can aid in disentangling the possibility of crossing-fiber effects; see following section.

From a theoretical standpoint, corticomotor projections were a hypothesized structure of interest. As described in Abercrombie et al. (2018), CORT increased activation in SMA/PMC and rescued depression-related deficits in memory formation for positive stimuli in severely maltreated women. On CORT day both sAA and rescue effects were significantly related to activation in these areas. No cortical regions innervated by IFOF fibers showed this group x drug interaction. And insofar as corticomotor and adrenal activity are related, projections via CST and other descending circuits would represent a parsimonious route.

### Possible sources of biological variation in corticomotor projection tract structure

Although the precise changes in axonal microstructure or fiber architecture cannot be resolved by FA (Jones et al., 2013b), interpretation depends at least in part on whether effects represent disrupted signaling or some other change in circuit function. Recently-published research (Mortazavi et al., 2018) suggests a possible, if highly speculative, mechanism to explain these effects. Histology of macaque CST found that, contrary to following smooth contours, many axons at the level of the centrum semiovale executed “microscopically sharp axonal turns and/or branches (radii ≤ 15 mm) into 2 sharply defined orientations, mediolateral and dorsoventral.” Further, high-angle diffusion MRI, which is capable of resolving crossing fiber orientation, was unable to distinguish between crossing fibers and sharp axonal turns.

The influence of intra-voxel discrepancy in fiber orientation on FA is thought to be large, relative to that of axonal physiology (Jones et al., 2013b). It is therefore conceivable that small variations in the proportion of descending fibers deflecting to mediolateral targets in this span of CST could yield statistically detectable changes in FA, even if restricted to a small population of axons bound for spinal circuits innervating sympathetic preganglionic cells. This would also be consistent with the fact that our FA effects were driven by changes in radial, rather than axial, diffusivity. Given the novelty of the above findings, further studies in animal models—which offer the additional advantage of well-characterized adverse caregiving paradigms—may serve to test the generalizability of our results, and fully elucidate any biological source of our structural findings.

### EA moderated associations between cluster FA and sAA

Under placebo, i.e., during basal levels of cortisol, the relation between SNS activation and bilateral corticomotor tract FA depended on severity of EA. Among participants with minimal history of childhood EA, FA was negatively associated with sAA, indicating that women with greater corticomotor white matter FA showed relatively lower SNS activation. For individuals with severe EA, however, results trended in the opposite direction such that higher white matter FA was weakly associated with relatively higher SNS activation.

These findings at baseline are interesting in light of recent nonhuman primate tracing studies indicating that spinal circuits influencing adrenal medulla are likely innervated by fibers from cortical motor areas (Dum et al., 2016). SMA was already known to be robustly associated with SNS tone and autonomic function (Medford and Critchley, 2010), consistent with its classical role preparing for strenuous action involving core musculature (Graziano, 2006); spinal projections therefore constitute a previously unknown, but logical, pathway with respect to modulation of peripheral stress-related activation. Previous analyses from this sample (Abercrombie et al., 2018), as well as other studies, have found functional alterations to SMA and adjacent premotor areas in individuals with history of maltreatment, especially in paradigms related to emotion regulation and executive control (for review, cf. McCrory et al., 2017). These alterations may also distinguish between individuals resilient to or at risk of psychopathology related to early adversity (Herringa, 2017). Taken together, one speculative interpretation might hold that history of exposure to EA alters the function of these areas and their projections to the SNS.

SMA and adjacent premotor areas, including dorsal cingulate, are known to integrate cognitive and affective inputs in motor planning (Picard and Strick, 2001; Shackman et al., 2011) along a continuous functional and physiological gradient: from anterior to posterior, cognitive processing converges on motor planning, and density of spinal cord-bound projections to adrenal medulla increase (Nakamura et al., 1998; Dum and Strick, 2002; Morecraft and Tanji, 2009). These systems appear to coordinate response selection and inhibition under complex, uncertain, and/or emotionally salient conditions (Nachev et al., 2008; McRae et al., 2010). However, despite their documentation in research concerning motor and some cognitive function, nomenclature for these areas varies in the emotion research discipline—perhaps due in part to limitations on spatial fidelity and resolution in functional imaging, or to challenges specific to more commonly-studied prefrontal association cortices. Study coordinates or clusters that overlap with preSMA, SMA, anterior PMC and other areas may be described using only directional or topographic terms: dorsomedial prefrontal cortex, superior frontal gyrus, dorsal anterior- or mid-cingulate, etc., whose boundaries may be defined differently across studies (see reviews: Ray and Zald, 2012; McCrory et al., 2017). It is possible that they may therefore play an under-recognized role linking emotion to behavior, particularly in the context of stress.

Furthermore, SMA in particular appears to facilitate voluntary emotion regulation (Buhle et al., 2014), alterations to which are considered a link between EA and risk of psychopathology (O'Mahen et al., 2015). Animal evidence for the role of these premotor circuits in inhibiting automatic and enabling intentional responses includes microstimulation of preSMA neurons (Isoda and Hikosaka, 2007), and macrostimulation in humans produces similar effects (Swann et al., 2012). A rodent paradigm found altered connectivity patterns in prefrontal and motor areas in female rats subjected to chronic social stress as pups (Nephew et al., 2017, 2018). Silvers et al. (2017) found that as typically-developing children reach adulthood, reactivity to aversive relative to neutral stimuli shifted from ventromedial (vmPFC) to dorsomedial prefrontal cortex (dmPFC), including preSMA, whereas Herringa and colleagues (Keding and Herringa, 2016; Wolf and Herringa, 2016) found a reverse profile of activation and limbic connectivity in children suffering from PTSD. In addition to SMA function, there is some independent evidence that CST structure is itself related to strategic aspects of emotional intelligence (Pisner et al., 2017).

That these systems should vary by exposure to early life adversity may represent divergent calibration of the neural circuitry supporting emotion regulation for vastly different environmental demands. Indeed, in a review of studies in EA populations, McCrory et al. (2017) found maltreatment-related effects on emotion regulation may persist in the absence of overt psychopathology, and suggest neural responses that are adaptive in an adverse caregiving environment confer “latent vulnerability” in adulthood. Other authors have converged on similar frameworks (e.g., Del Giudice et al., 2011; Blair and Raver, 2012; Teicher and Samson, 2013).

### CORT eliminated effects of EA on relations between cluster FA and sAA

While EA moderated the relation of corticomotor tract FA and SNS activation under baseline conditions, CORT administration eliminated these associations. Following acute cortisol elevation SNS tone may depend on neural pathways other than corticomotor projections. There is a relative dearth of studies directly examining effects of GCs on neural control of SNS; however, GCs affect a variety of structures that regulate SNS output, including the hypothalamus, amygdala, and bed nucleus of the stria terminalis (Ulrich-Lai and Herman, 2009). Research in rodents implicates amydalofugal pathways during GC elevation: whereas GCs exert negative feedback on the HPA axis in hypothalamus and cortical areas, they increase CRH in the amygdala. This affects fear-related behaviors associated with SNS activity, such as startle (Erickson et al., 2003). Indeed, a study (Song et al., 2014) specifically examining FA in tracts connecting to brainstem nuclei found depression-related alterations not in CST, but in the solitary tract, which is known to reciprocally connect to amygdala; however, they did not report on alterations due to EA. Future research is needed to determine the neural circuitry through which GCs interact with SNS function, and how circuits are affected by EA. This is relevant as evidence suggests that in some disorders, such as PTSD, there are alterations in the relation between GCs and SNS (Yehuda et al., 1998).

### Implications for etiology and treatment of stress-related psychopathology

Our findings lend further evidence to a large body of literature showing that history of maltreatment is an important etiological factor in depression and other forms of psychopathology, which has bearing on treatment selection (Nemeroff et al., 2003; Williams et al., 2016). Furthermore, early adversity alters stress response systems, and alterations in cortisol signaling and HPA regulation are more often observed in depressed adults with vs. without history of childhood adversity (Heim et al., 2008; Abercrombie et al., 2018). The current findings extend upon these literatures by implicating corticomotor projections as part of a stress responsive neural circuit whose functioning is associated with variation in early caregiving.

These premotor areas and their influence on SNS function may represent an important target for clinical intervention. In addition to their involvement in emotion regulation—an important buffer to psychopathology, as discussed in the section above, “EA moderated associations between cluster FA and sAA”—recruitment in medial premotor areas may play a compensatory role in individuals with maltreatment and/or psychiatric disorders according to several lines of evidence, including a meta-analysis (Herringa et al., 2016; McTeague et al., 2017). Corticomotor projections influencing SNS could comprise a neural circuit important for psychotherapeutic benefit in individuals with history of adverse caregiving.

This circuit may therefore be an important target for behavioral, pharmacological, or neuromodulatory interventions. It could play a role in effective treatments such as behavioral activation (Dimidjian et al., 2011; Ekers et al., 2014), aerobic exercise (Rebar et al., 2015; Schuch et al., 2016), or yoga (Pascoe and Bauer, 2015; Cramer et al., 2017). Our prior research may also suggest that interventions targeting cortisol signaling could operate in part through activation of SMA and PMC (Abercrombie et al., 2018). Repetitive Transcranial Magnetic Stimulation (rTMS) to dmPFC reduced depression severity (Downar et al., 2014), although this was applied to an area anterior to preSMA, was effective specifically in patients with lower anhedonia symptomatology, and initial replication efforts have not been successful (Bakker et al., 2015). Gutman et al. (2009) performed probabilistic structural connectivity for a target of deep brain stimulation (DBS) previously found effective in treating depression, located in the anterior limb of the internal capsule (IC), and its connectivity patterns bear some superficial resemblance to those of our TBSS-derived cluster, although of course anterior to our cluster intersecting the posterior limb of IC. Though evidence is mixed, cortical motor circuits may represent targets for psychiatric treatment with neuromodulation, possibly in combination with context-specific cognitive therapy related to emotion regulation. Based on findings suggesting that functioning within these circuits is related to early experience, effectiveness of such treatments may vary based on history of childhood adversity.

As mentioned previously, a “mismatch” may arise between phenotype—which developed in response to a stressful early environment—and post-developmental context, that increases risk of depression or other psychopathology. Progress in understanding how to re-align phenotype and environmental demands may improve the specificity of treatments beyond that achieved by accounting for early experience alone.

### Limitations

As this is a cross-sectional study, we are unable to draw firm conclusions as to how childhood EA altered developmental trajectories leading to adult differences in brain structure and stress neuromodulator function. In both the current analyses and in fMRI analyses in the same sample (Abercrombie et al., 2018), we found that EA but not depression severity was associated with neural alterations. However, due to the relatively low rate of PTSD in this sample, we are unable to examine neural correlates related to PTSD.

Another limitation of our study is that we were not able to recruit a sample of adults who experienced extreme EA but who do not exhibit psychopathology, and for statistical reasons we needed to exclude a single participant who presented with extreme EA but no depressive symptoms. Future research should endeavor to include such participants with targeted recruiting to allow investigation of the role of EA in stress-related physiology along a full range of severity of psychopathology.

In addition, emerging methods for disambiguating crossing fibers may aid in establishing the exact contributions of each tract captured by our clusters of interest. Further study comparing these methods against histological studies are vital for interpretation. Finally, the present study investigates the effects of exogenous cortisol; future research must also elucidate the impact of naturalistic environmental stressors on relations among white matter structure, cortisol, and SNS activation.

## Conclusion

We found that history of emotional abuse—but not severity of depression—moderated relations among corticomotor white matter structure and SNS activation in a sample of women with levels of depression along a broad continuum of severity. The findings may suggest that such pathways supporting neural influence on SNS activity vary based on prior experience of adverse caregiving. Furthermore, cortisol administration abolished these associations, consistent with evidence that cortisol acutely alters neural systems supporting the regulation of peripheral stress physiology.

Functional systems-based approaches to the etiology of mental disorders (Insel, 2014) offer insights into biological mechanisms that may be affected by life history, and which can be targeted for clinical applications. Disruptions to both SNS and HPA responses to stress are candidate systems in stress-related disorders such as depression and PTSD. Cortical motor systems and their efferent fibers, whose outputs appear to ultimately modulate adrenal medulla (Dum et al., 2016), may represent a neural circuit affecting alterations in stress response systems. Future studies should further characterize the action of this pathway under different neural, endocrine, and cognitive conditions, and test whether such action differs according to exposure to adverse caregiving and other forms of early life adversity.

## Author contributions

HA designed the study and obtained funding. HA, EW, RH, and RB refined the design and implemented the study. EW and RH programmed the picture viewing task. EW and RB programmed scan parameters. EW, RH, CF, and HA collected data. EW and RH organized and cleaned data. EW, CF, and HA analyzed data. CF processed and analyzed neuroimaging data in consultation with RB, MM, and HA. CF wrote the first draft of the manuscript in consultation with MM and HA. HA wrote sections of the manuscript. CF, RH, and HA composed figures and tables. All authors read and approved the manuscript.

### Conflict of interest statement

The authors declare that the research was conducted in the absence of any commercial or financial relationships that could be construed as a potential conflict of interest.
