# Supplementary material for: Childhood Emotional Abuse Moderates Associations Among Corticomotor White Matter Structure and Stress Neuromodulators in Women With and Without Depression
Source: Front Neurosci. 2018 Apr 23;12:256. doi: 10.3389/fnins.2018.00256 (PMC5925965; doi:10.3389/fnins.2018.00256)
Supplement: Supplementary file 1 [file DataSheet1.docx]

**Childhood Emotional Abuse Moderates Associations Among Corticomotor White Matter Structure and Stress Neuromodulators in Women With and Without Depression**

**Supplementary Materials**

**Participant Inclusion and Exclusion Criteria**

*Inclusion Criteria*

- Female
- 18 to 45 years of age
- English fluency
- Able to lie still on their back for up to 90 minutes
- Willing and able to return for all visits
- Able to provide written informed consent prior to participation
- In good physical health as determined by medical history
- If a nicotine user, able to refrain from nicotine use for 2 hours prior to fMRI scanning and throughout the scan visits
- Additional criteria for never-depressed subjects: Free of current or past DSM-5 diagnoses of MDD or other depressive disorders
- Additional criteria for subjects with current or past depression: Meets DSM-5 criteria for a current or past depressive disorder diagnosis

*Exclusion Criteria*

- Lifetime history of psychosis or mania
- Substance use disorder within the last 6 months
- Significant risk for suicide
- Claustrophobia
- Acute or chronic physical (non-psychiatric) illness
- Daily nicotine use
- Use of antidepressants, other psychotropic medication, or medications that alter glucocorticoids
- Hormonal contraceptives
- Peri- or postmenopausal signs
- Highly irregular periods
- Pregnancy or breastfeeding within the last 6 months
- Illicit drug use within the last 4 weeks (verified by negative urinary drug test)


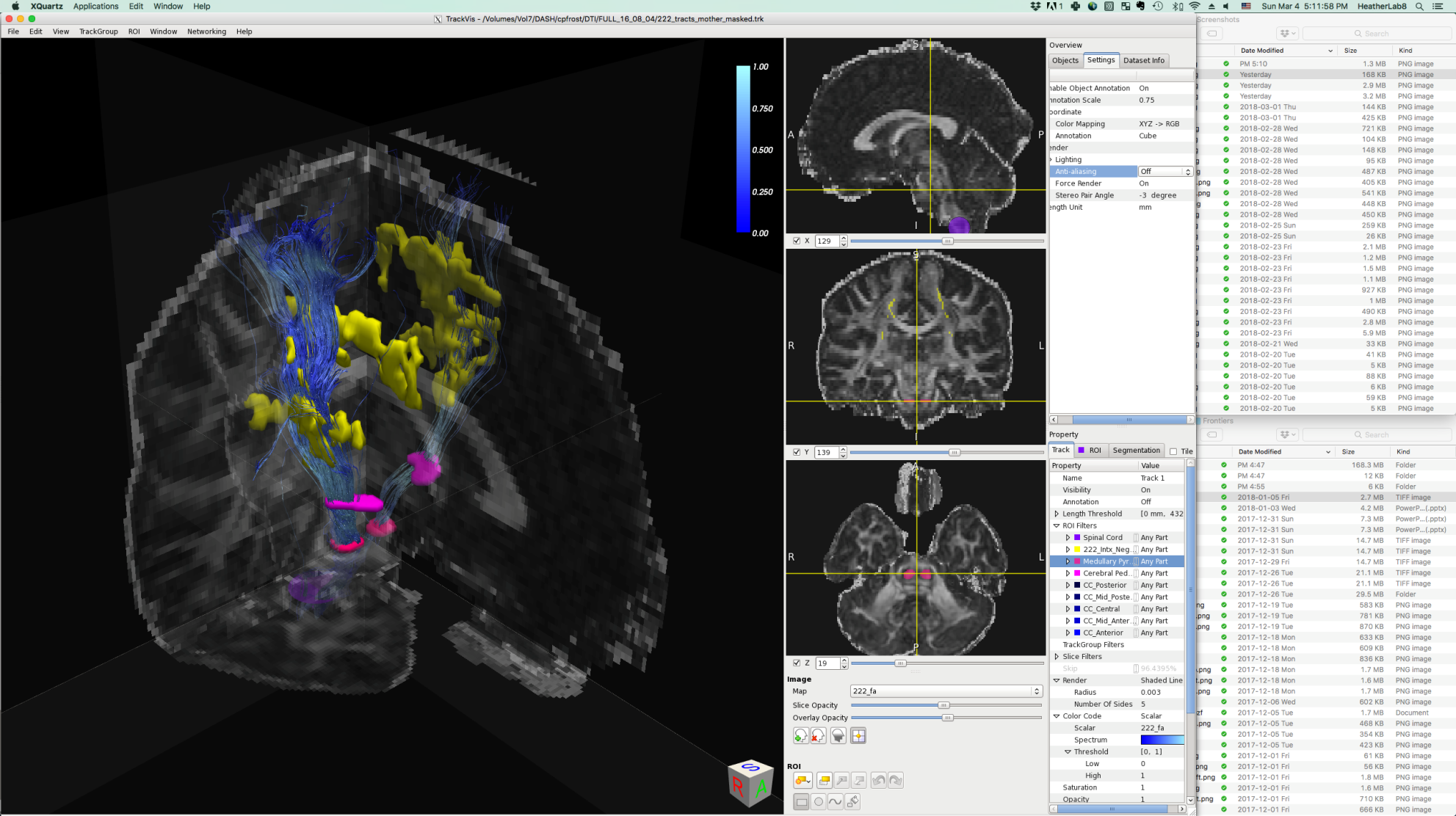

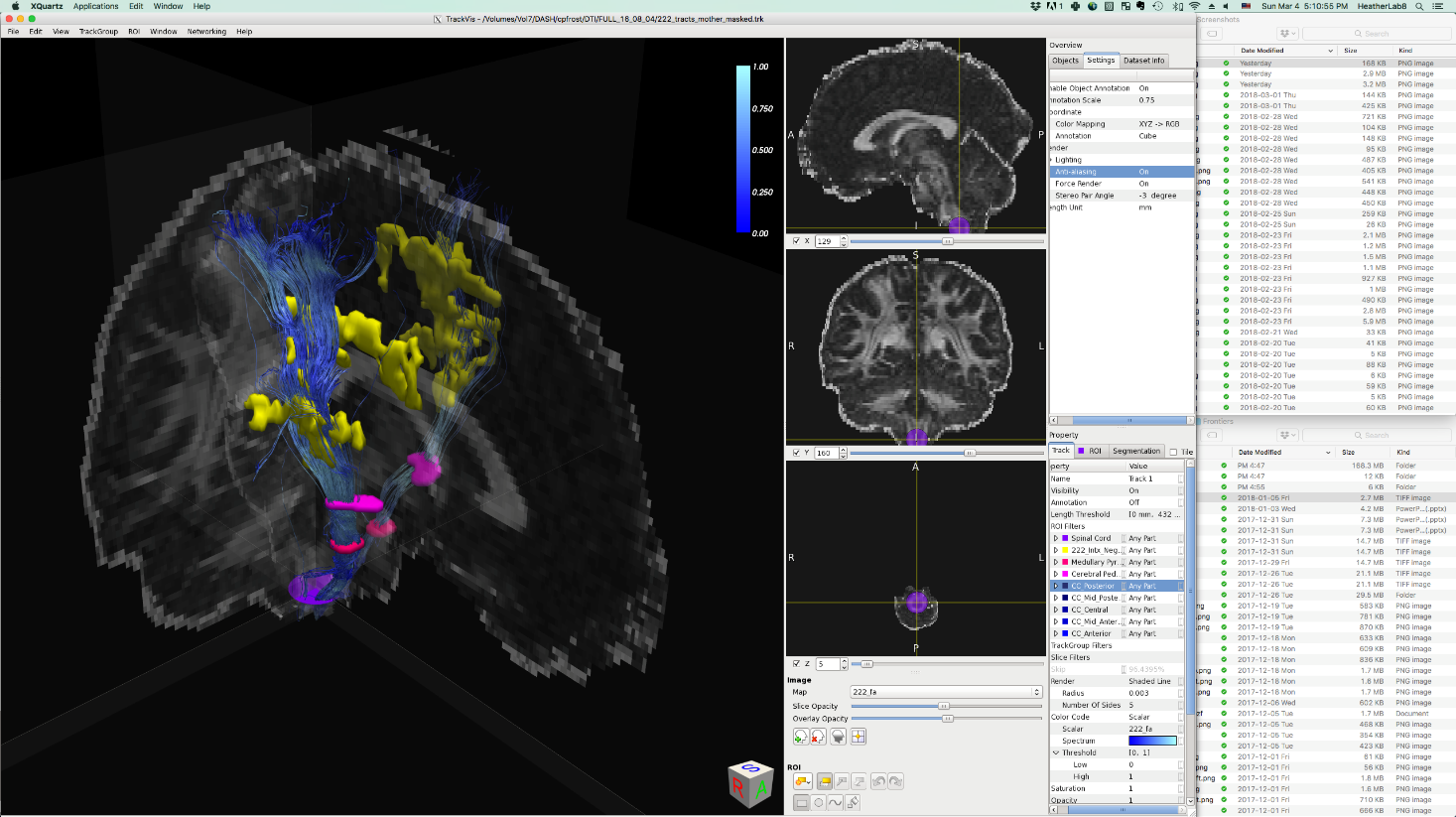
**Figure S1.** **ROI-based tractographic selection of Corticospinal Tract (CST).** A) CST was identified as passing through several obligatory ROIs, traced on axial slices in TrackVis. These included B) the cerebral peduncles at the level of the mammillary bodies, C) the pyramidal tracts at the level of the medulla, and D) a disk of 6mm radius encompassing the spinal cord at a slice inferior to the cerebellum. In addition, fibers that intersected the corpus callosum were excluded.


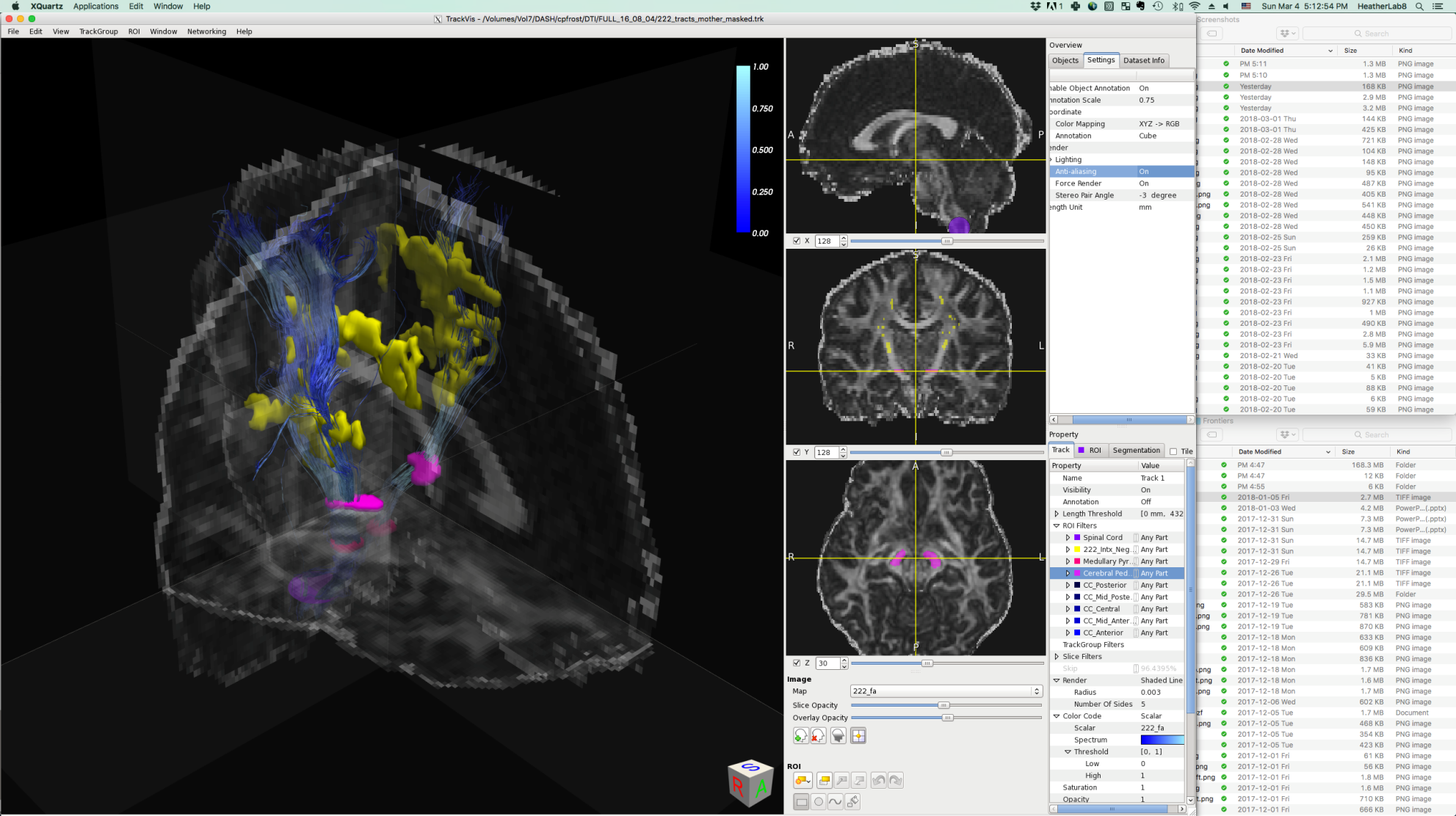

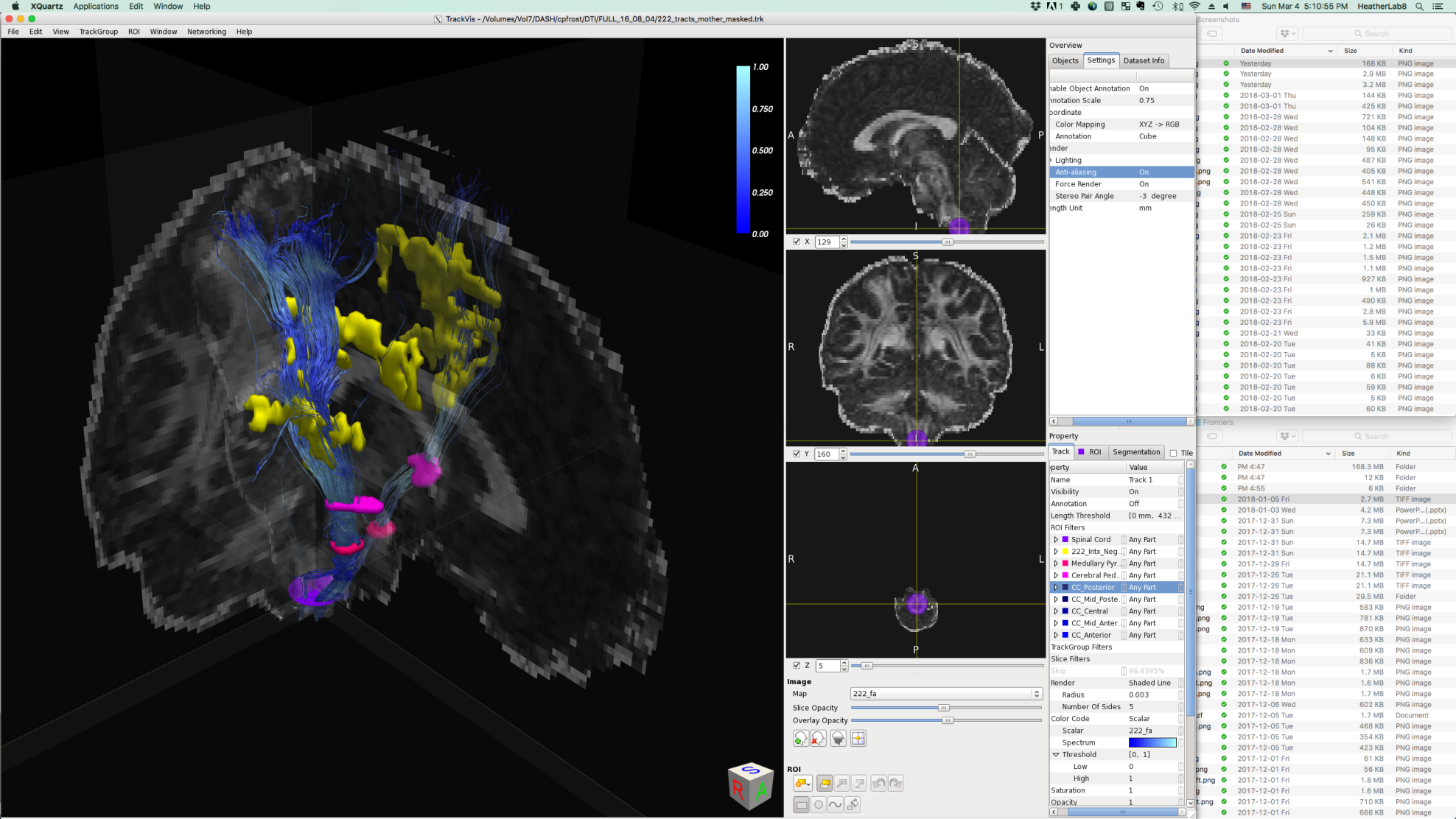
A B C D

**Figure S2. Clusters and tracts in which depression severity is related to fractional anisotropy (FA).** No clusters were related to either sAA_CORT-Placebo_ or its interaction with depression severity.


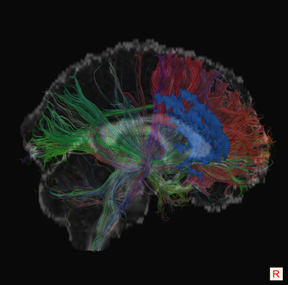

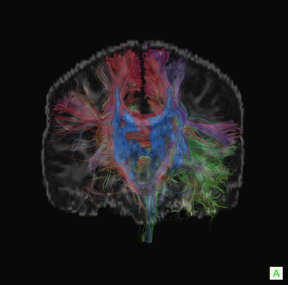

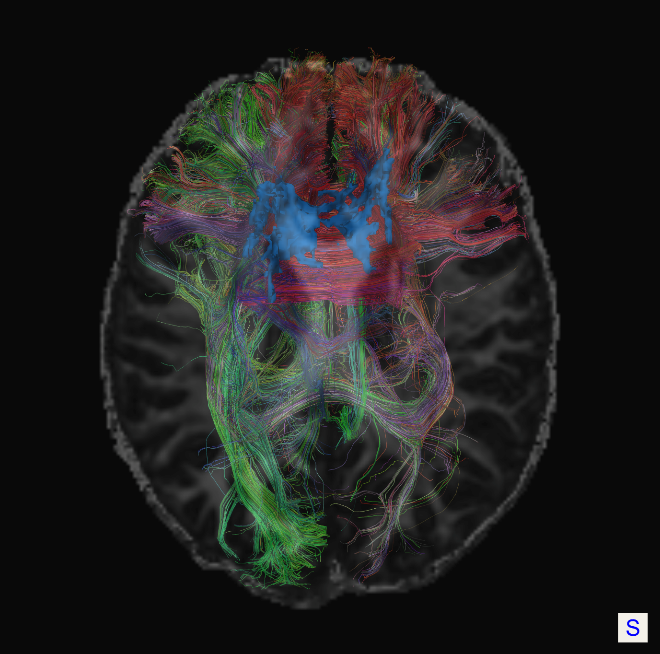


**Figure S3. Streamlines traversing clusters and reaching spinal cord, unconstrained by cortical terminations.**


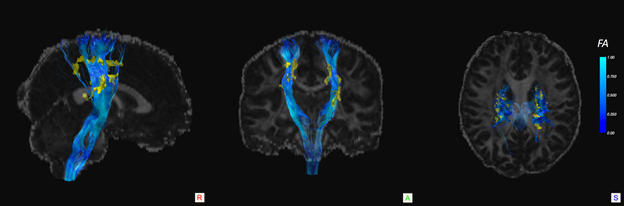


**Table S1. Depression severity and fractional anisotropy (FA).** Significant clusters associated with depression severity.

| **Cluster** | **Volume (voxels)** | **Coordinate (peak)** | **Coordinate (CM)** | **Stat (peak)** | ***P* Value (corrected)** |
| --- | --- | --- | --- | --- | --- |
| Anterior CC | 2672 | (-16,42,1) | (-2,40,-4) | t = 3.48 | .01 |
| Left ALIC | 438 | (-26,44,-7) | (-23,45,-9) | t = 3.16 | .04 |
| Right CS | 239 | (18,43,11) | (18,43,11) | t = 2.25 | .04 |
| Left external capsule | 70 | (-29,33,-18) | (-28,34,-15) | t = 3.79 | .04 |

CM, center of mass; CC, corpus callosum; ALIC, anterior limb of the internal capsule; CS, centrum semiovale.
